# Chronic larval exposure to thiacloprid impairs honeybee antennal selectivity, learning and memory performances

**DOI:** 10.3389/fphys.2023.1114488

**Published:** 2023-04-20

**Authors:** Li Ke, Xiasang Chen, Pingli Dai, Yong-Jun Liu

**Affiliations:** ^1^ State Key Laboratory of Resource Insects, Institute of Apicultural Research, Chinese Academy of Agricultural Sciences, Beijing, China; ^2^ Key Laboratory of Pollinating Insect Biology, Institute of Apicultural Research, Chinese Academy of Agricultural Sciences, Beijing, China

**Keywords:** *Apis mellifera* L., antenna, electroantennography, thiacloprid, learning and memory

## Abstract

The use of agricultural neonicotinoid insecticides has sub-lethal chronic effects on bees that are more prevalent than acute toxicity. Among these insecticides, thiacloprid, a commonly used compound with low toxicity, has attracted significant attention due to its potential impact on the olfactory and learning abilities of honeybees. The effect of sub-lethal larval exposure to thiacloprid on the antennal activity of adult honeybees (*Apis mellifera* L.) is not yet fully understood. To address this knowledge gap, laboratory-based experiments were conducted in which honeybee larvae were administered thiacloprid (0.5 mg/L and 1.0 mg/L). Using electroantennography (EAG), the impacts of thiacloprid exposure on the antennal selectivity to common floral volatiles were evaluated. Additionally, the effects of sub-lethal exposure on odor-related learning and memory were also assessed. The results of this study reveal, for the first time, that sub-lethal larval exposure to thiacloprid decreased honeybee antenna EAG responses to floral scents, leading to increased olfactory selectivity in the high-dose (1.0 mg/L) group compared to the control group (0 mg/L *vs*. 1.0 mg/L: *p* = 0.042). The results also suggest that thiacloprid negatively affected odor-associated paired learning acquisition, as well as medium-term (1 h) (0 mg/L *vs*. 1.0 mg/L: *p* = 0.019) and long-term memory (24 h) (0 mg/L *vs*. 1.0 mg/L: *p* = 0.037) in adult honeybees. EAG amplitudes were dramatically reduced following R-linalool paired olfactory training (0 mg/L *vs*. 1.0 mg/L: *p* = 0.001; 0 mg/L *vs*. 0.5 mg/L: *p* = 0.027), while antennal activities only differed significantly in the control between paired and unpaired groups. Our results indicated that exposure to sub-lethal concentrations of thiacloprid may affect olfactory perception and learning and memory behaviors in honeybees. These findings have important implications for the safe use of agrochemicals in the environment.

## Introduction

Insects play a critical role in agriculture as pollinators, contributing to ecosystem stability through their pollination of crops and cultivated and wild plants (Mashilingi et al., 2022). The widespread use of synthetic chemicals like pesticides, particularly neonicotinoids, is considered one of the most determinant factors in the decline of pollinator populations worldwide (Rundlöf et al., 2015; Janousek et al., 2023). Neonicotinoids are the most widely used pesticides globally and are used to control a variety of sucking pests (Elbert et al., 2008; Xu et al., 2022). Residues of these insecticides have been found not only in soil (Liu Z. K. et al., 2022) and water (Mahai et al., 2019) but have also been traced in the pollen and nectar due to their systemic properties (Singla et al., 2020; Alkassab et al., 2023) and can harm non-target pollinator honeybees by acting on nicotinic acetylcholine receptors (nAChRs) in the insects’ nervous system (Tomizawa and Casida, 2003) and non-neuronal ACh system (Grunewald and Siefert, 2019). In recent years, neonicotinoid residues have been discovered in wild and managed bees and their honey samples worldwide (Mrzlikar et al., 2019; Tang et al., 2019; Wang et al., 2020; Kavanagh et al., 2021).

Prior studies have extensively investigated the negative impacts of sub-lethal doses of neonicotinoid insecticides on honeybees (Blacquiere et al., 2012; Singla et al., 2020), including physiological changes (Catae et al., 2018; Roat et al., 2020; Lv et al., 2023), delayed development (Li et al., 2022), weakened immune response (Chmiel et al., 2019; Annoscia et al., 2020; Zhang et al., 2022), and impaired colony reproduction (Schott, et al., 2021). Specifically, neonicotinoids disturb honeybee behaviors, including waggle dancing (Tison et al., 2020), foraging and homing (Capela et al., 2022), and colony performance (Negi et al., 2022; Reiner et al., 2022). These impacts interact with honeybee pathogens (deformed wing virus, Israeli acute honeybee paralysis virus, etc.) and parasites (*Varroa destructor*, for instance), synergistically affecting the immune and detoxification abilities of honeybees (Annoscia et al., 2020; Parekh et al., 2021).

Most previous research has focused on the effects of nitro-substituted neonicotinoids (imidacloprid, clothianidin, and thiamethoxam) on honeybees, with relatively few studies investigating the impact of cyano-substituted neonicotinoids such as thiacloprid. Thiacloprid is commonly applied to flowering crops like oilseed rape, fruits (Piechowicz et al., 2021), vegetables, and grains (Xu et al., 2023), which are significant sources of honey pollen for honeybees in China (Piechowicz et al., 2021; Wen et al., 2021). Recent investigations have suggested that thiacloprid is less toxic to honeybees than nitro-substituted neonicotinoids (Decourtye and Devillers, 2010; Manjon et al., 2018), possibly due to its metabolism by cytochrome *P450*-dependent monooxygenases (Haas et al., 2022). Thiacloprid is detoxified by *CYP9Q3* in honeybees (Manjon et al., 2018) and bumblebees (Troczka et al., 2019) as well as by *CYP9BU* in *Osmia bicornis* solitary bees. However, despite its comparatively lower toxicity, exposure to thiacloprid has shown detrimental effects on honeybees, including reduced survival rates (Liu et al., 2019), decreased immunity (Shi et al., 2018; Mollmann and Colgan, 2022; Orčić et al., 2022), impaired learning and memory, impaired foraging and homing behavior (Fent et al., 2020; Begna and Jung, 2021), altered circadian and sleep patterns (Tasman et al., 2021), damaged spermatogenesis (Hartman et al., 2021), interactions with *Nosema ceranae* and black queen cell virus (BQCV) (Doublet et al., 2015), and changes in social communications (Tison et al., 2016).

Flower volatiles and pheromones perception, as well as olfactory learning and memory, are critical for honeybee behaviors such as foraging (Paoli and Galizia, 2021). Odors are detected by the olfactory receptors (ORs) on honeybee antennae, which are subsequently processed in the antennal lobes (AL) glomeruli and transferred to the mushroom bodies (MB) *via* projection neurons. The MB are the higher brain areas of honeybees (insects) that integrate information from all sensory modalities and are involved in memory generation and storage (Mariette et al., 2021).

Previous studies have shown that neonicotinoids, as an agonist of nAChRs at the postsynaptic membrane, interfere with neuronal signal transduction to disrupt honeybee (*A. mellifera* L.) odor perception in the antennal lobe (Andrione et al., 2016; Mustard et al., 2020). In the Kenyon cells (KCs) of the honeybee MB, neonicotinoids evoke sustained depolarization, causing KCs to not respond to acetylcholine, which may be the primary cause of honeybee learning and memory disorders (Palmer et al., 2013). The knockout of Dα1 and Dβ2 nAChR in the fly MB recapitulated neonicotinoid-induced memory impairment (Tasman et al., 2021). However, studies on the higher olfactory pathways in honeybees (AL and MB) have not addressed whether the observed effects are derived from the perireceptors in the honeybee antennae, which are the major sensory sensilla of the insect olfactory system. Compared to mosquitoes and flies, honeybees have fewer odorant binding proteins (21 OBPs) but more olfactory receptors (Robertson et al., 2003; Larter et al., 2016; Wheelwright et al., 2021). These receptors are distributed among ∼60,000 olfactory receptor neurons (ORNs) in the antennal bulb (Paoli and Galizia, 2021). Honeybee antennal electroantennography (EAG) responses to flower volatiles and pheromonal compounds vary with neonicotinoids in a dose-dependent manner (Favaro, et al., 2022). Additionally, odor learning and memory modulated the plasticity of olfactory receptors (MaBouDi et al., 2017).

The present study evaluated the effect of thiacloprid on antennal activities and olfactory selectivity, as well as learning and memory. We exposed honeybee larval stages to thiacloprid (0.5 mg/L and 1.0 mg/L) in in the laboratory to simulate chronic and cumulative neonicotinoid toxicity in the honeybee hive. EAG recordings and odor-associated learning and memory behaviors tests were used to assess the effects. Our results indicated that thiacloprid exposure during the larvae stage decreased overall adult honeybee EAG responses to floral scents, resulting in increased olfactory selectivity and impaired adult honeybee learning acquisition and memory abilities, which may partially contribute to the impairment of antennae odor receptors. This study is the first to report the chronic and cumulative effects of thiacloprid exposure during larval development on honeybee antennae.

## Materials and methods

### Animal samples

Honeybees (*A. mellifera* L.) were collected from three colonies at the apiary at the Institute of the Apicultural Research, CAAS (40°1′29″N, 116°16′51″E) during the spring and summer seasons when the hives were free of visible mites (*V. destructor*), and insecticide sprays are prohibited in the nearby nectar sources within an approximate radius of 10 km. The colonies were identified to be free of bacterial diseases (American and European foulbrood), fungal diseases (Nosema, chalkbrood, and stonebrood), and viruses (deformed wing virus and acute honeybee paralysis virus) *via* PCR testing. The larvae were reared according to a standard protocol described by Schmehl et al. (Schmehl et al., 2016), which has been widely employed in numerous studies (Chen et al., 2021; Değirmenci et al., 2022; Netschitailo et al., 2023). We confirmed the dependability of this larva-rearing approach, as we did not observe queen or queen-like honeybee emergence despite administering royal jelly to larvae aged >3 days. To initiate the experiment, a honeybee queen was confined within a queen excluder push-in cage (13 × 16 cm) and placed on an empty comb in the center of a hive. After 24 h, the queen was released, and the comb was left in the hive for 3 days during the egg stage until the larvae hatched. On the 4th day, the comb was transferred to the laboratory, and the newly hatched larvae were transferred to sterilized 48-well culture plates. The larvae were then cultured in an incubator (35°C, ∼94% R.H.) for 6 days, during which time they were fed either a control diet or a diet containing thiacloprid (0.5 mg/L, 1.0 mg/L). The feeding was discontinued during the pupae stage, and the larvae were raised in the incubator (35°C, ∼75% R.H.) for 12 days. The total thiacloprid dose ingested by the larvae was 0.08 μg/bee and 0.16 μg/bee in the 0.5 mg/L and 1.0 mg/L treatment groups, respectively, based on the volume of diet consumed over the 6-day period. The sub-lethal dose of thiacloprid used in our experiment was determined based on previous studies (Tison et al., 2016; Shi et al., 2018). After the adult honeybees emerged, they were reared on 50% sucrose (w/w) in wooden cages, after which their learning and memory abilities were tested (Denton et al., 2021) Electroantennography recording on honeybee antennae were conducted 7–8 days after emergence, from May to September 2022.

### Chemical compounds and preparations

Thiacloprid (catalog 37905–100 mg-R, Sigma-Aldrich) was dissolved in dimethyl sulfoxide (DMSO, MP Biomedical) and then diluted in sterilized water to make a 250 mg/L stock solution. During the laboratory larval feeding period, the thiacloprid stock solution was further diluted to 0.5 mg/L and 1.0 mg/L using a freshly prepared larvae diet consisting of royal jelly, glucose, fructose, yeast extract, and water. The proportions of each component varied by larvae age. The larvae diets were prepared 1 day in advance and stored at −20°C. For the *in vitro* larval feeding stage, the diets were thawed at 9:00 a.m. by placing them in an incubator at 35°C for approximately 0.5 h. The highest DMSO concentration used in the study (0.01%) was below the reported toxic dose for honeybees (Milchreit et al., 2016). Thus, we did not set up a solvent-control group for further experiments.

### Electroantennography recordings

Electroantennography (EAG) was employed to record electrical potentials transmitted from honeybee antennae to the brain in response to various odors. This method is commonly used to study insect olfactory perception (Hummel and Miller, 1984). To minimize variability in sensitivity, EAG signals were recorded from the right antenna of 7-day-old adult honeybees in each treatment group (0 mg/L, 0.5 mg/L, and 1.0 mg/L). The excised antenna was clamped to the two distal fertilizing electrodes with conductive gel (REF15-60, Sigma, United States). Seven different odors include four floral scents (nonanal, isoamyl acetate, geranylic acid, and 1-hexanal), two bee pheromones (citral and 1-hexanol), and a negative control odor (mineral oil). Mineral oil is a colorless and odorless liquid that is used to establish a baseline measurement of electrical activity in the absence of an odor stimulus in EAG. Comparing the response to mineral oil with that of an odor stimulus helps to ensure the validity and reliability of the EAG results. Each odor was presented to each antenna in a fixed order, with each odor replicated three times during the EAG recording. We used floral scents (Sandoz, 2011; Balbuena and Farina, 2020), alarm pheromone (Wager and Breed, 2000; Wright et al., 2015), and aggregation pheromone (Roussel et al., 2014). Specific information on the seven odors is included in Table 1. These odors were diluted in mineral oil to a concentration of 1% (v/v). In each test, 10 μL of the diluted solution was deposited on filter paper (0.6 cm × 4 cm) and inserted into a Pasteur pipette. The pipette tip was then connected to the circulating airflow tube. A stimulus controller (CS-55, Syntech, Kirchzarten, Germany) delivered a humidified continuous airflow at 20 mL/s, with the odor stimuli presented as 0.5 s pulses at 10 mL/s airflow. A 30 s interval was inserted between each stimulation to allow for baseline restoration. The EAG measurements were performed using an Intelligent Data Acquisition Controller (IDAC-2-USB, Syntech, Kirchzarten, Germany). The signals were amplified by a 10 × AC/DC headstage preamplifier (DTP-1, Syntech, Kirchzarten, Germany) and recorded using Gc-Ead 1.2.5 software (Syntech, Kirchzarten, Germany). The recording was carried out at a sample rate of 200 Hz and the signals were then filtered using a 10 Hz high-pass filter. The signals were analyzed offline using Gc-Ead 1.2.5. The amplitudes of the signals were calculated as the peak value subtracted from the baseline value. Ten biological replicates were performed for EAG recordings for each treatment group (0 mg/L, 0.5 mg/L, and 1.0 mg/L). To evaluate the changes in the baseline EAG responses for each antenna, positive (1-hexanal) and negative (mineral oil) control odors were used.

**TABLE 1:** Registration information for the odor stimuli used in the experiments.

Odor	Floral	Pheromone	Chemical abstracts service (CAS) number	PubChem compound identification (CID) number	Reference
Nonanal	√		124-19-6	31289	Sandoz (2011), Balbuena and Farina (2020)
Isoamyl acetate	√		123-92-2	31276	Sandoz (2011), Balbuena and Farina (2020)
Geranylic acid	√		459-80-3	9989	Sandoz (2011), Balbuena and Farina (2020)
1-Hexanol		√	111-27-3	8103	Wager and Breed (2000), Wright et al. (2015)
Citral		√	5392-40-5	638011	Roussel et al. (2014)
1-Hexanal	√		66-25-1	6184	Sandoz (2011), Balbuena and Farina (2020)
R-Linalool	√		78-70-6	6549	Claudianos et al. (2014))
Mineral oil			8042-47-5		Liu J. J. et al. (2022)

### Olfactory learning and memory behavioral evaluations

We tested the olfactory-associated learning acquisition and memory recall behaviors in 7-day-old adult honeybees exposed to thiacloprid during the larval stage. As the olfactory system does not fully develop until 2–3 days after emergence from the comb (Denton et al., 2021), before the experiment, individual honeybees were habituated to the setup for 2 h, with the head restrained to a cylindrical copper tube and the abdomen free to move. Furthermore, honeybees that did not respond to the 30% sucrose (w/w) solution were eliminated from further testing. The learning and memory behaviors tests were conducted using a self-developed odor generator synchronized to an automatic sucrose-feeding device. This device enabled the precise control of odor and sucrose delivery for behavior evaluation. Honeybees from each treatment group were divided into paired and unpaired training groups, in which the paired group received a 6 s R-linalool odor (diluted in mineral oil to 1% v/v) paired with a drop of 50% sucrose as a reward for the last 3 s of R-linalool odor presentation. The unpaired group, on the other hand, only received the 6 s R-linalool odor without a sucrose reward. For each training session, the proboscis extension reflex (PER) response was recorded. The learning acquisition training was repeated 10 times, at 5-min intervals, in both paired and unpaired training groups. After completing the conditioned olfactory learning acquisition training, we investigated whether the associated linalool odor elicited PER memory retention at 1 h (medium-term memory) and 24 h (long-term memory) (Menzel, 1999). A total of 355 honeybees were used in the learning and memory behavior tests (*N* = 58–60).

To determine how thiacloprid exposure during the larval stage affects the EAG recordings of adult bee antennae following olfactory conditioning behavior training, we made recordings from 10 antennae in each group after a 24 h long-term memory test that used the same conditioned R-linalool odor, as well as mineral oil. As described previously, each antenna was fixed and stimulated with 1% R-linalool. The peak EAG responses were averaged over three repetitions, and the mean peak EAG amplitudes for each group were calculated.

### Data and statistical analysis

#### EAG response analysis

To determine the odor selectivity of the antennae, the peak amplitudes of EAG responses to seven stimulated odors were plotted as the average of the three replicates. We arranged the EAG peak response of each antenna to the seven odors, with the greater amplitude in the center and lower amplitude on the sides. Then, to determine the odor selectivity, the responses were fitted to a Gaussian curve using the following equation:

$$y=y_{o}+\frac{A}{\omega \sqrt{\pi /2}}e^{-2\frac{{\left(x-x_{c}\right)}^{2}}{\omega^{2}}},$$

where $y_{o}$ is the baseline of the Gaussian distribution, A is the peak of the response dataset, $x_{c}$ is the center of the response, and *ω* is the tuning width of the fitting curve. Two parameters were calculated to represent the selectivity of antennae to different odors: the half- width at half-height (HWHH) of the Gaussian fitted curve and the odor stimulus selectivity index (SI). The SI represents the amplitude difference between the maximum and minimum values of the odor response curve and was calculated as SI = [a − (b + c)/2]/[a + (b + c)/2], where a is the highest EAG amplitude in the aforementioned Gaussian fitted curve and b and c are the bilateral lowest values of the Gaussian fitting curve (Gittelman, et al., 2012). A higher HWHH value indicates poor odor selectivity and *vice versa*. Larger SI index values indicate stronger odor selectivity.

#### Statistical analysis

**FIGURE 1:**
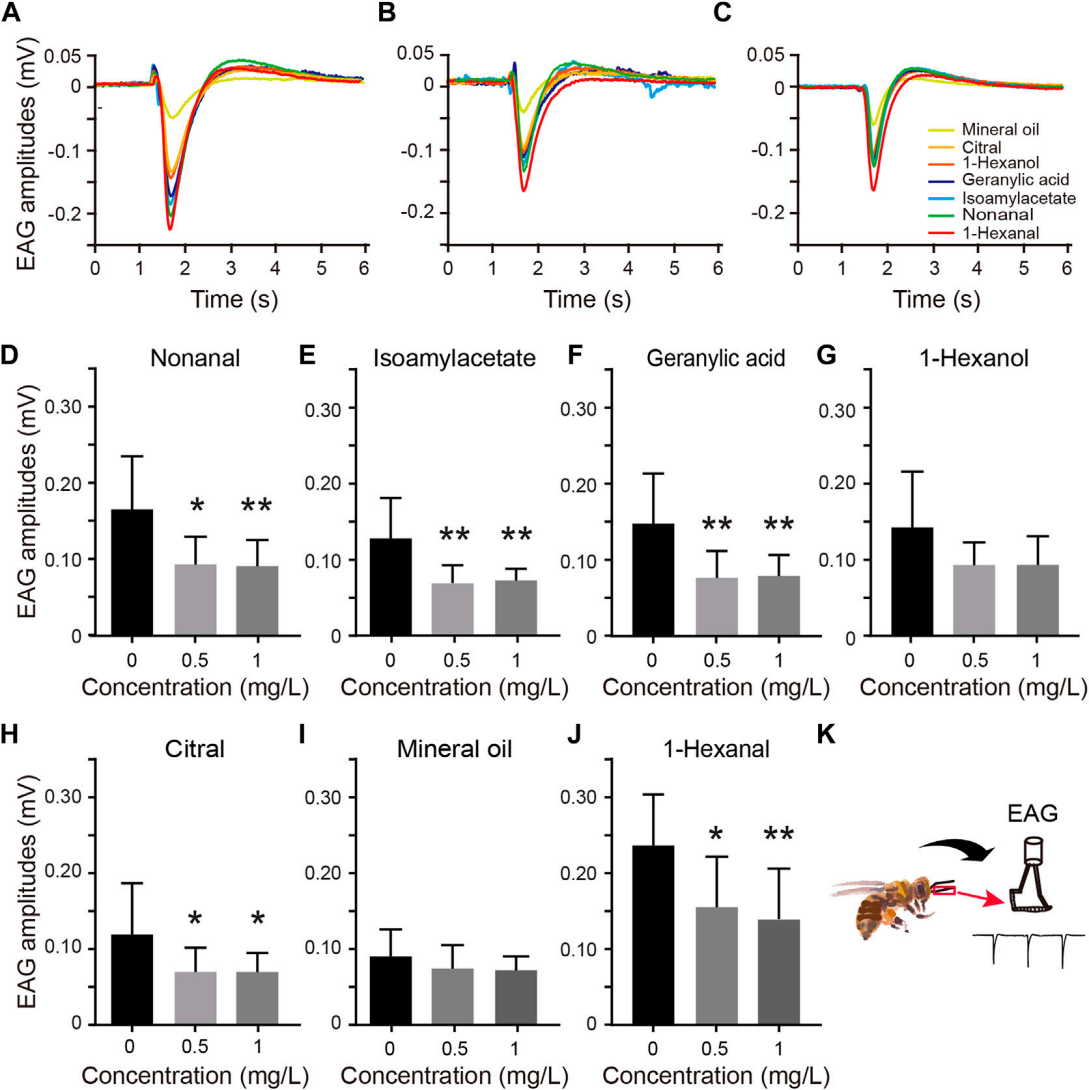
Electroantennography (EAG) amplitudes of odor-evoked responses in newly emerged honeybees exposed to thiacloprid during the larval stage. **(A–C)** Amplitude plot of the averaged EAG responses of three representative honeybee antennae to seven different floral odors at varying thiacloprid treatment concentrations (0 mg/L, 0.5 mg/L, and 1.0 mg/L). **(D–J)** Odor-evoked EAG responses of all antennae to nonanal, isoamyl acetate, geranylic acid, 1-hexanol, citral, mineral oil, and 1-hexanal. The data are displayed as means ± SEM, *N* = 10 bees in each treatment group, with three technical replicates. Statistical analyses were performed using one-way ANOVA followed by Dunnett’s test, *p < 0.05, **p < 0.01. **(K)** Schematic diagram of EAG records of honeybee right antennae.

**FIGURE 2:**
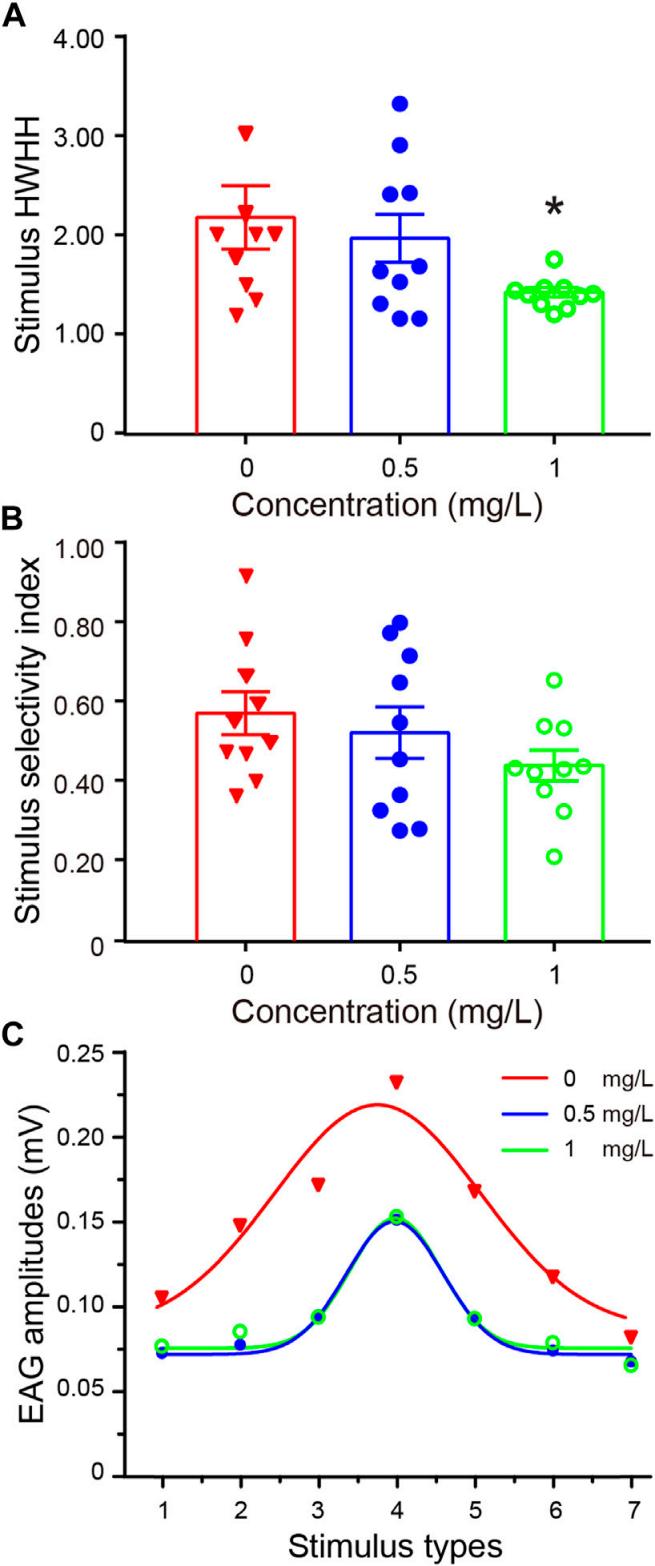
Distributions of olfactory selectivity for each treatment group. **(A)** Mean olfactory tuning width (half-width at half-height of the waves, HWHH) distributions. The HWHH in the 1.0 mg/L thiacloprid treatment group is significantly lower than that of the control group (Kruskal–Wallis followed by Dunn’s test, *p* = 0.042). The error bars are SEM, *N* = 10 bees in each treatment group, with three technical replicates. **(B)** Average olfactory selectivity index (SI) across all treatment groups (mean ± SEM). The treatment groups showed no significant differences (one-way ANOVA followed by Dunnett’s test, 0.5 mg/L *vs*. 0 mg/L, *p* = 0.747; 1.0 mg/L *vs*. 0 mg/L, *p* = 0.165). **(C)** Gaussian-fit curves of all antennae responses to seven odor responses in three treatment concentrations (0 mg/L, 0.5 mg/L, and 1 mg/L).

**FIGURE 3:**
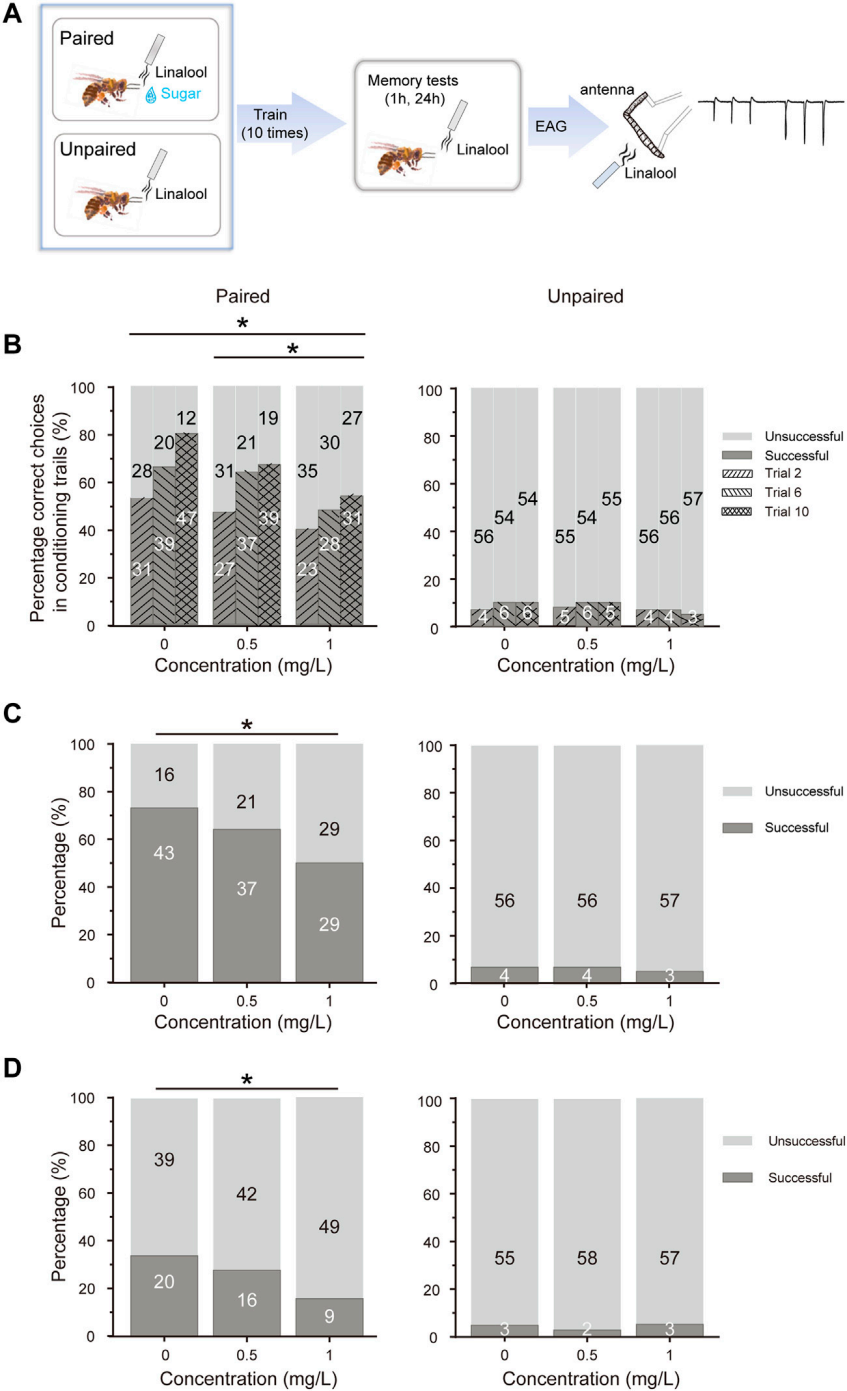
Thiacloprid exposure negatively impacts learning acquisition and memory retention. **(A)** Schematic representation of olfactory learning and memory behaviors, as well as electroantennography (EAG) recordings, following training. The paired group received R-linalool as a conditioned stimulus in conjunction with a sucrose reward, whereas the unpaired group also received R-linalool, but without the sugar reward. The learning acquisition was repeated 10 times, and the memory recall was tested for 1 h (medium-term memory) and 24 h (long-term memory) after learning acquisition. After the memory retention testing sessions, EAG recordings were made from the trained honeybee antenna. **(B)** Honeybee learning acquisition was influenced by three thiacloprid concentrations. The percentages of correct choices for the paired (left panel) and unpaired (right panel) groups were plotted as training sessions 2, 6, and 10 from each group. *N* = 58 bees in the paired group, and *N =* 60 bees in the unpaired group. **(C,D)** Memory retention performance represented by recall success for the paired (left panel) and unpaired (right panel) groups in 1 h (medium-term) and 24 h (long-term) memory at three treatment concentrations (0 mg/L, 0.5 mg/L, and 1 mg/L). Chi-squared test, with stars indicating statistical differences between groups. *p < 0.05, **p < 0.01.

Data were analyzed using the Shapiro–Wilk normality test to verify the normal distribution before performing the statistical analyses. One-way ANOVA was performed, followed by multiple comparisons using Dunnett’s test to compare the EAG peak amplitudes of antennae to seven odors (Figure 1), and SI values (Figure 2B) across treatment groups. Kruskal–Wallis tests with Dunn’s multiple comparison test were used to compare HWHH values (Figure 2A). Chi-squared tests were conducted to analyze behavioral data, including correct memory recall percentages (Figures 3B–D) to assess differences across treatments. The EAG amplitudes after R-linalool learning in each treatment group were compared using Kruskal–Wallis tests with Dunn’s tests. Moreover, pairwise *t*-tests were conducted to compare EAG amplitudes between paired and unpaired groups for each treatment. Statistical analysis was performed using GraphPad Prism (GraphPad Prism 7.0, United States). *p* < 0.05 was considered statistically significant.

## Results

### EAG responses of honeybee antennae to different odors

The schematic diagram of the EAG recording setup for honeybee antennal is shown in Figure 1K. Due to the distinct diversity of olfactory receptors, the antennas’ sensitivity to different odors varied. The EAG responses of honeybee antenna to seven distinct odor stimuli (floral scents and pheromones) were recorded separately for each treatment group. Figures 1A–C illustrate the EAG response waveforms of honeybee antennae to these seven odor stimuli at varying thiacloprid exposure concentrations (0 mg/L, 0.5 mg/L, and 1.0 mg/L). The results showed an overall decrease in EAG amplitudes in response to different odors among treatment groups. Next, we compared all antennae response amplitudes for those odors in each treatment group. The population results are shown in Figures 1D–J, in which the antennae EAG responses to various odors show consistent declines among the three concentrations. As shown in Figures 1D–J, the EAG response amplitude of antennae to four floral scents (nonanal, isoamyl acetate, geranylic acid, and 1-hexanal)odors were generally lower in the thiacloprid-treated groups than those in the control group. The EAG responses to nonanal (Figure 1D), isoamyl acetate (Figure 1E), geranylic acid (Figure 1F), and 1-hexanal (Figure 1J) differed significantly between the thiacloprid-treated (1.0 mg/L) and control groups (one-way ANOVA followed by Dunnett’s test, *p* = 0.001, *p* = 0.003, *p* = 0.007, *p* = 0.006). Likewise, in the 0.5 mg/L thiacloprid-treated group, the EAG amplitudes to two floral scents (isoamyl acetate and geranylic acid) decreased significantly compared to the control group (*p* = 0.002, *p* = 0.004), while the other two floral scents (nonanal and 1-hexanal) showed significant differences between the 0.5 mg/L-treated and control groups (*p* = 0.011, *p* = 0.016). However, the EAG response to the pheromone 1-hexanol (Figure 1G) was similar across groups, while the EAG response to the citral pheromone (Figure 1H) showed significant differences between thiacloprid-treated (0.5 mg/L and 1.0 mg/L) and control (0.5 mg/L vs. 0 mg/L: *p* = 0.041; 1.0 mg/L *vs*. 0 mg/L: *p* = 0.047) groups. We compared the EAG responses to specific odors with that of the negative control odor (mineral oil) (Supplementary Figure S1A). The results suggested that, among the tested odors, the EAG amplitudes of 1-hexanol and 1-hexanal were significantly higher in all treatment groups (0 mg/L, 0.5 mg/L, and 1.0 mg/L) compared to mineral oil (paired *t*-test, *p* < 0.05). The EAG responses to nonanal and isoamyl acetate were significantly higher than those to mineral oil in the 0 mg/L (*p* < 0.01) and 1.0 mg/L groups (*p* < 0.05), but not in the 0.5 mg/L group. Finally, the EAG response to geranylic acid and citral was only significantly greater than mineral oil at the 0 mg/L concentration (geranylic acid, *p* < 0.01; citral, *p* < 0.05). Overall, the EAG response to specific odors was generally higher than that to mineral oil, which also indicated the reliability of our EAG recording throughout the experiments. In summary, thiacloprid exposure during the larval stage significantly decreased the overall EAG responses to both floral scents and pheromones in adult honeybees.

### Olfactory selectivity of honeybee antennae

While the EAG amplitudes to various odors differed, we next assessed the selectivity toward various odors. We rearranged the EAG amplitudes to a sequence of high values in the middle and low values on the bilateral, followed by Gaussian fitting. The half-width at half-height (HWHH) of the waves and the selectivity index (SI) were calculated as the parameters to describe the selectivity of odors. The HWHH value, which depicted the sharpness of the waves, indicated that greater and lower values represented broader and narrower odor selectivity, respectively.

The data from 10 antennas of each group showed a significant decrease in HWHH, with the average HWHH decreasing 32% from the 1.0 mg/L treatment group to the control group, indicating that thiacloprid induced sharper olfactory selectivity at the highest treatment concentration (1.0 mg/L), as shown in Figure 2A (Kruskal–Wallis followed by Dunn’s test, 1.0 mg/L *vs*. 0 mg/L, *p* = 0.052; 0.5 mg/L *vs*. 0 mg/L, *p* > 0.5). Furthermore, the HWHH differences between the low-dose (0.5 mg/L) and control groups, as well as the high-dose (1.0 mg/L) and 0.5 mg/L groups were not significant. These findings suggest that thiacloprid treatment reduced EAG amplitudes to odors in honeybee antennae and reinforced the selectivity of odors, as reflected in the reduced HWHH value in the treatment groups compared to the control groups. In addition to HWHH, olfactory selectivity was evaluated by SI. The results showed no significant difference between treatment groups (Figure 2B; one-way ANOVA followed by Dunnett’s test, 1.0 mg/L *vs*. 0 mg/L, *p* = 0.747; 0.5 mg/L *vs*. 0 mg/L, *p* = 0.165). The average olfactory EAG responses of all tested antennae to the seven odors in each treatment group are plotted in Figure 2C, withthe responses fitted to the Gaussian curve. The results showed that the HWHH of the curve was significantly narrower in the thiacloprid treatment groups than in the control group. Although the highest and lowest differences in EAG reactions (SI values) were similar across different treatment groups, this was consistent with the findings in Figures 2A, B. In Figures 2B, C, the influence of thiacloprid on the narrower odor selectivity is similar. Thiacloprid inhibited the antennal potential, with a stronger effect on non-dominant odors. This resulted in higher selectivity for specific odors (1-hexanal in our experiments) with a smaller HWHH in the treatment group.

### Learning and memory behaviors of adult bees

We investigated honeybee odor-associated learning acquisition and memory recall for each treatment group. In the learning training session, a paired group in which the conditioned odor stimulus of R-linalool was paired with a sucrose reward, and an unpaired group where R-linalool odor was not associated with a reward (Figure 3A). The memory recall tests were conducted 1 h and 24 h after the 10 learning trials. As shown in Figure 3B, the accuracy of olfactory learning in the paired group of each treatment group increased with an increasing number of training trials. Moreover, thiacloprid showed a dose-dependent effect on the PER correct choices ratios in the paired conditions. In the treatment groups, honeybees exposed to 1.0 mg/L and 0.5 mg/L of thiacloprid as larvae showed significantly lower olfactory paired learning acquisition compared to the control honeybees (0 mg/L *vs*. 0.5 mg/L: *p* = 0.181; 0 mg/L *vs*. 1.0 mg/L: *p* = 0.001; 0.5 mg/L *vs*. 1.0 mg/L: *p* = 0.024). However, the unpaired group showed no differences in the PER correct choice ratios in Figure 3B across treatment groups (chi-squared test, 0.5 mg/L or 1.0 mg/L *vs*. control, *p* = 0.999; *p* = 0.317). Thus, honeybee larvae exposed to thiacloprid showed reduced olfactory-associated learning performance as adult honeybees, which may affect their foraging activity.

After 1 h (medium-term memory) and 24 h (long-term memory) of the last learning acquisition trial, the efficacy of honeybees’ memory recall to R-linalool odor was evaluated. The successful recall of R-linalool odor was determined through the honeybees’ PER responses. The ratio of successful memory recall tests after 1 h and 24 h for paired and unpaired training honeybees is plotted in Figures 3C, D. In the medium-term memory (1 h) recall test, approximately 70% of the control honeybees in the paired group successfully remembered the R-linalool with a sucrose reward, while 90% of the unpaired group showed unsuccessful PER responses. The proportion of individuals in the paired group that successfully remembered the odor was significantly lower in the 1.0 mg/L treatment group compared to the control group (chi-squared test, χ^2^ = 5.54, df = 1, *p* = 0.019). While the low-dose (0.5 mg/L) group showed a slight decrease compared to the control, it was not statistically significant (*p* = 0.391). Similarly, for the long-term memory tested at 24 h after learning acquisition, the success rate of memory recall tests for the paired training group decreased across all treatment groups, with only 30% of honeybees in the control group showing successful recall. Compared to the control, the proportion of honeybees in the 1.0 mg/L group that remembered R-linalool odor was significantly lower (*p* = 0.037). However, the memory recall did not differ significantly between the treatments for unpaired honeybees in either the medium-term (1 h) (Figure 3C right panel) or the long-term (24 h) memory tests (Figure 3D) (0.5 mg/L or 1.0 mg/L *vs*. 0 mg/L, *p* = 0.62; *p* = 0.966).

In short, thiacloprid exposure during the larval stage had a detrimental impact on adult honeybee learning and medium-term and long-term memory. This effect was dose-dependent, with higher treatment concentrations resulting in greater negative effects on learning and memory compared to lower concentrations.

### EAG response of adult honeybees to R-linalool learning

**FIGURE 4:**
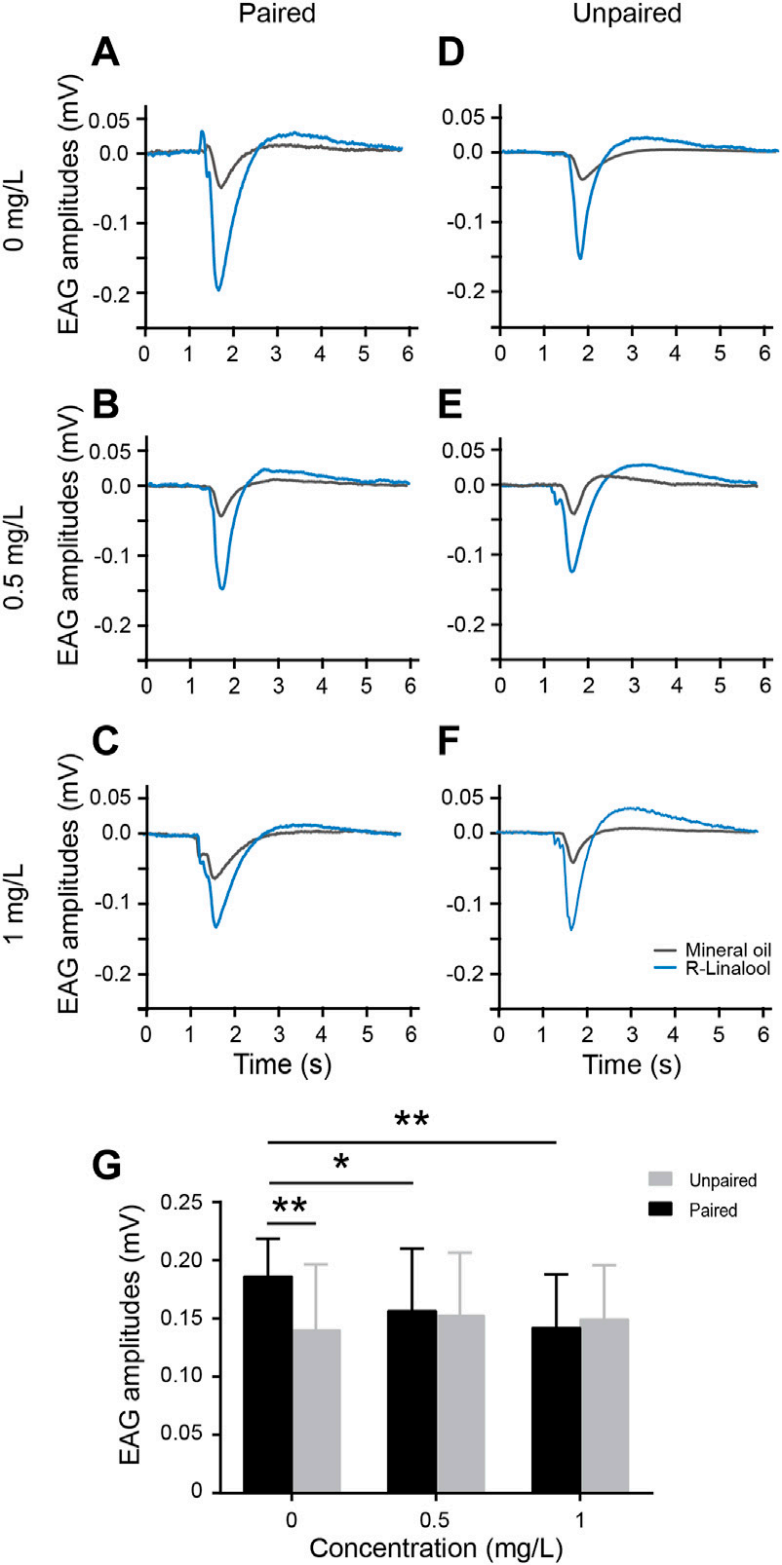
Electroantennography (EAG) recordings following learning acquisition training. **(A–F)** Example EAG response waves of antennae from the paired and unpaired groups (average of three replicates), using mineral oil as a negative odor control (gray curves). **(G)** Average EAG peak amplitudes in the paired and unpaired groups for three treatment groups (0 mg/L, 0.5 mg/L, and 1 mg/L) (mean ± SEM, *N* = 10 bees in each group with four technical replicates). Kruskal–Wallis test followed by Dunn’s and pairwise *t*-tests. *p < 0.05, **p < 0.01.

As noted previously, thiacloprid exposure in the honeybee larval stage negatively affects the learning and memory of adult honeybees after emergence. We next investigated the effects of this exposure on antennae, as the frontmost olfactory receptors, by assessing their EAG response to R-linalool-associated-olfactory learning. We performed odor-conditioned training in paired and unpaired groups, with 10 trials conducted as described previously. Twenty-four hours after the final learning acquisition session, the right antenna of each honeybee was dissected for EAG recording. Individual antennae were stimulated three times for 0.5 s each with the conditioned odor R-linalool. Each group contained ten antennae. Figures 4A–F show typical EAG waves 24 h after conditioned odor training in the paired and unpaired groups across three concentration treatment groups. The EAG amplitudes of R-linalool odor in the examples ranged mostly between 0.1 and 0.2 mV, while the mineral oil control remained stable within 0.03–0.05 mV. Similar to the pattern of different thiacloprid concentrations on antennae EAG response before conditioned R-linalool learning, the antennae activity amplitudes decreased as thiacloprid exposure concentration increased following odor conditioning. In contrast, the EAG peak amplitudes after conditioned odor trials were comparable across treatments in the unpaired group, differing from the overall pattern shown in Figure 1. The average EAG peak amplitudes for the population of antennae from both paired and unpaired groups in each treatment group are plotted in Figure 4G, with similar patterns observed in population data to individual antennae EAG results. Thiacloprid significantly decreased the EAG peak amplitudes in response to R-linalool odor after 24 h of the conditioned training, compared to the control group. We observed a statistically significant reduction in EAG activity between the 1.0 mg/L high-dose and control groups (Kruskal–Wallis followed by Dunn’s test, *p* = 0.001), as well as between the 0.5 mg/L and control groups (*p* = 0.027). In contrast, the unpaired group did not show a decreasing trend across treatments. The average amplitudes were 0.14 ± 0.06 mV for the control group, 0.15 ± 0.05 mV for the 0.5 mg/L thiacloprid-treated group, and 0.15 ± 0.05 mV for the 1.0 mg/L thiacloprid-treated group. However, the paired group exhibited a different pattern. In the control condition, the peak EAG amplitudes to R-linalool in the paired group were significantly greater than those in the unpaired group (paired *t*-test, *p* = 0.001). However, in the 0.5 mg/L and 1.0 mg/L thiacloprid treatment groups, the peak EAG amplitudes did not differ significantly between the paired and unpaired groups (*p* = 0.73, *p* = 0.712). This suggests that thiacloprid may affect the functioning of the antennae odor receptors and impair odor perception during conditioned training, which could partially contribute to the impairment of memory recall. We also compared the EAG amplitudes of R-linalool in the paired and unpaired groups to mineral oil, as shown in Supplementary Figure S1B. The EAG response to mineral oil was significantly lower than R-linalool for all three treatment concentrations in the paired (paired *t*-test, *p* = 0.001) and unpaired groups (*p* = 0.001).

## Discussion

The negative effects of neonicotinoid insecticides on honeybees have been investigated in several studies (Annoscia et al., 2020; Tasman et al., 2021; Zhang et al., 2022). Thiacloprid impairs perception, learning, and memory in adult honeybees (Tison et al., 2016). However, little is known about the effects of thiacloprid on olfactory perireceptors in the honeybee antennae. In this study, we investigated the impact of chronic exposure to different concentrations of thiacloprid during the larval stage on the olfactory antennae EAG response and learning and memory in worker honeybees. The results showed that exposure to sub-lethal doses of thiacloprid during the larval stage partially reduced adult honeybee EAG responses to floral scents and pheromones, resulting in greater antennal odor selectivity. Furthermore, thiacloprid treatment negatively affected learning and medium-term and long-term memory. Additionally, thiacloprid treatment significantly altered the EAG response of adult honeybees to R-linalool following learning acquisition training.

### Antennal olfactory selectivity decreased by reducing EAG response amplitudes

The results of the present study provide evidence that thiacloprid exposure during the honeybee larval stage affected the antennal EAG amplitudes in *A. mellifera* L. for typical floral volatiles; specifically, the olfactory response to nonanal, isoamyl acetate, geranylic acid, and 1-hexanal odors decreased significantly between the treatment (0.5 mg/L and 1.0 mg/L) and control groups. The results of this study indicated that thiacloprid exposure in the larval stage of honeybees interfered with antennal olfactory selectivity in adult honeybees. It is crucial for honeybees to detect floral scent compounds, which helps them to find host plants (Değirmenci et al., 2022). A decreased selectivity toward floral volatiles might lead to problems in locating host plants and might affect the foraging efficiency (Tison et al., 2016) or colony development of bees (Ellis et al., 2017). Our results are consistent with previous studies of the ablation of honeybee ability to discriminate and associate floral scents by acute treatment with neonicotinoid thiamethoxam (Mustard et al., 2020) and decreased antennal sensitivity to 2-phenylethanol in *O. bicornis* and *Bombus terrestris* due to clothianidin exposure (Straub et al., 2021). However, we detected no reduction in antennal response to the 1-hexanol alarm pheromone, which is involved in nestmate recruitment. Conversely, another aggregation pheromone, citral, showed a notable reduction in EAG amplitudes between treatment and control groups. This finding is consistent with that of a study by Straub et al. (2021), which showed that the neonicotinoid clothianidin had a similar diminished effect on pheromone ethyl palmitate EAG perception in bumblebees (*B. terrestris*). The reason for the lack of significant effect of thiacloprid treatment on the antennae EAG response to 1-hexanol could be due to the differential responsiveness among scent receptor classes, which highlights the need for further investigation into a broader range of pheromone scent classes.

Analysis of the selectivity of antennae EAG response to various odor stimuli (HWHH analysis) revealed that thiacloprid increased the olfactory selectivity of honeybees to specific floral odors in the 1.0 mg/L treatment group (Figure 2A) due to a stronger inhibition effect on non-dominant odors. Olfactory selectivity is an essential aspect of honeybee foraging and communication with mates. The capacity of honeybees to distinguish different floral scents and pheromones in the environment allows for accurate communication with their peers (Paoli and Galizia, 2021). The volatile content in flowers affects the frequency of honeybees and bumblebee visits to tomatoes (Liu J. J. et al., 2022). In addition, evidence indicates that neonicotinoid pesticides impair bees’ ability to discriminate floral scents (Mustard et al., 2020). Our results demonstrated that exposure to thiacloprid during the honeybee larval stage impacted olfactory selectivity, leading them to be more selective for specific scents. This may partly explain the reduction in honeybee foraging efficiency (Colin et al., 2019; Tasman et al., 2020) and social communication (Tison et al., 2016; Straub et al., 2021) caused by neonicotinoid use.

We discovered that thiacloprid treatment in honeybee larvae reduced the amplitude of EAG in adult honeybees. However, the mechanisms behind this phenomenon remain unclear based on the EAG recordings in this study. Because EAG signals represent the summed electrically potential changes in olfactory receptors on the honeybee antennae, the thiacloprid-induced variations of the EAG amplitude may be due to alternations in either the number of activated olfactory receptors or the activation strength of single neurons through changes in the firing rate. Honeybees (*A. mellifera* L.) possess 170 annotated olfactory receptor genes and 21 odorant binding proteins, primarily located in the antennae (Foret and Maleszka, 2006). Previous studies reported that exposure to neonicotinoids, such as imidacloprid and thiamethoxam, can lead to significant reductions in odor-binding protein expression in *Apis cerana* (Li, et al., 2015), as well as odorant receptor genes (Roat et al., 2020).

### Impairments of olfactory-associated learning and memory recall

To investigate the long-term effect of larval thiacloprid treatment on adult honeybees, we evaluated the honeybees’ odor-associated learning acquisition and memory recall activities. We discovered that larval exposure to thiacloprid reduced adult honeybee PER acquisition rates associated with paired odor. However, we observed no significant effect in the unpaired group (Figure 3B). In addition, honeybee medium-term (Figure 3C) and long-term (Figure 3D) memory recall capacities were disrupted in the paired group treated with thiacloprid. The unpaired group did not display any PER in memory recall tests as the odor was not coupled with the sugar water reward, thus preventing memory formation. These results are similar to those of previous studies showing that acute or chronic exposure to neonicotinoids impaired medium- and long-term olfactory memory (Tison et al., 2016; Tison et al., 2017). Imidacloprid contamination of *A. mellifera* L. (Delkash-Roudsari et al., 2020) or *A. cerana* (Tan et al., 2015) larvae reduced olfactory associative behaviors of honeybee workers.

The neural mechanism underlying the impairment of learning and memory in bees is due to neonicotinoids acting as antagonists of nicotinic acetylcholine receptors (nAChRs). nAChRs play crucial roles in neuronal plasticity, brain development (Cecchini and Changeux, 2022), and the formation of learning memory in honeybees (Cano-Lozano et al., 1996; Grunewald and Siefert, 2019). Chronic exposure to neonicotinoids caused nAChR desensitization (Mustard et al., 2020), altered odors encoding information (Ohlinger et al., 2022), and reduced microglomerular density, which are responsible for olfactory learning and memory functions (Peng and Yang, 2016; Tavares et al., 2019).

### Changes in EAG response after olfactory conditioning training

The EAG recordings of antennal activity after odor-associated learning acquisition training showed decreased activity of the paired groups due to the thiacloprid administration. Although slight increases in antennal response amplitudes were observed in unpaired groups with increasing thiacloprid concentrations, this trend was not statistically significant (Figure 4). Notably, the control treatment group showed a substantial elevation in EAG response to conditioning (paired group) odor, but not to simple odor exposure (unpaired group). This phenomenon may be akin to odor sensitization, which is classically described as an initial stage of olfactory memory formation (Menzel, 1999). However, this sensitization was absent in the thiacloprid-exposed groups, which may account for their poorer learning performance. The EAG findings may contribute to the observed alterations in memory recall, as evidenced by the decreased memory recall in the paired odor-associated training groups but not in the unpaired groups (Figures 3C, D). This suggests that thiacloprid may reduce the expression of antennae odor receptors, which could be influenced by neonicotinoids (Li, et al., 2017) and pre-odor experiences (Marachlian et al., 2021). The effect observed in the periphery antennae may stem from feedback from the antennal lobe (Jernigan et al., 2020) or higher brain centers such as the mushroom bodies. Therefore, some of the defects in olfactory learning and memory caused by thiacloprid may originate from the disruption of odor coding in honeybee antennae.

## Conclusion

The results of our study demonstrated the effects of thiacloprid exposure during the larval stage of honeybees (*A. mellifera* L*.*) on the olfactory responses of adult honeybees. We observed significant decreases in the amplitudes of the antennal responses to various floral scents and the pheromone citral. To evaluate the effects of thiacloprid on honeybee olfactory selectivity, we applied Gaussian fitting of the EAG amplitudes. The results revealed a higher selectivity with smaller HWHH for specific odors. Furthermore, thiacloprid impaired learning acquisition, as well as medium-term (1 h) and long-term memory (24 h) in adult honeybees. This was evidenced by a decreased EAG response following the learning of floral odor R-linalool. These findings suggest that exposure to thiacloprid during the larval stage can disrupt odor coding of the peripheral antennae of the emerged honeybees, contributing to impaired odor perception, olfactory learning, and memory. These results have important implications for the safety of agrochemical use in the environment.

## Data Availability

The raw data supporting the conclusion of this article will be made available by the authors, without undue reservation.
