# Supplementary figures and images for: Chronic larval exposure to thiacloprid impairs honeybee antennal selectivity, learning and memory performances
Source: Front Physiol. 2023 Apr 20;14:1114488. doi: 10.3389/fphys.2023.1114488 (PMC10157261; doi:10.3389/fphys.2023.1114488)

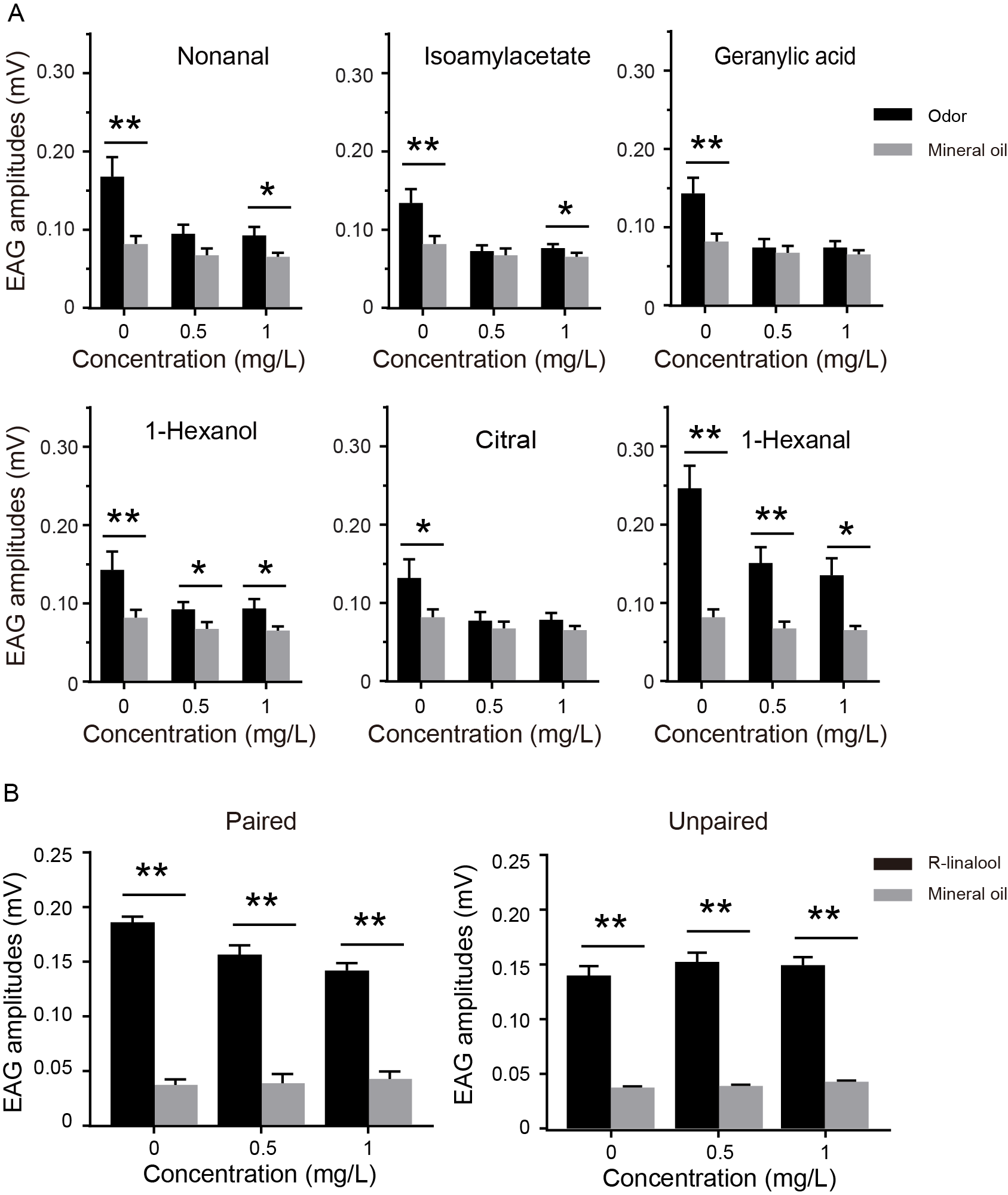

Supplement: Supplementary file 1 [file Image1.tif]
